# System Dynamics of Cognitive Vulnerabilities and Family Support Among Latina Children and Adolescents

**DOI:** 10.1007/s10567-022-00395-3

**Published:** 2022-03-04

**Authors:** Peter S. Hovmand, Esther J. Calzada, Lauren E. Gulbas, Su Yeong Kim, Saras Chung, Jill Kuhlberg, Carolina Hausmann-Stabile, Luis H. Zayas

**Affiliations:** 1 School of Medicine, Case Western Reserve University, Cleveland, USA; 2 Steve Hicks School of Social Work, The University of Texas, Austin, USA; 3 Department of Human Ecology, The University of Texas, Austin, USA; 4 SKIP, Washington University, St. Louis, USA; 5 System Stars, LLC, St. Louis, USA; 6 Graduate School of Social Work and Social Research, Bryn Mawr College, Bryn Mawr, USA

**Keywords:** Cognitive vulnerabilities, Suicidal ideation, Latina youth, Dynamical systems, Feedback theory, System dynamics, Loop dominance, Loop scores

## Abstract

The paper describes an approach to developing a data-driven development of a feedback theory of cognitive vulnerabilities and family support focused on understanding the dynamics experienced among Latina children, adolescents, and families. Family support is understood to be a response to avoidant and maladaptive behaviors that may be characteristic of cognitive vulnerabilities commonly associated depression and suicidal ideation. A formal feedback theory is developed, appraised, and analyzed using a combination of secondary analysis of qualitative interviews (*N* = 30) and quantitative analysis using system dynamics modeling and simulation. Implications for prevention practice, treatment, and future research are discussed.

**Supplementary Information:**

The online version contains supplementary material available at 10.1007/s10567-022-00395-3.

## Introduction

Latinx youth are at disproportionate risk for depression and suicidality (Baca-Garcia et al., 2010; Centers for Disease Control & Prevention, 2016; Polo & Lopez, 2009; Rew et al., 2001; Romero, 2014; Tortolero & Roberts, 2001). As toddlers (Weiss et al., 1999) and preschoolers (Calzada et al.,^ 2015^), Latinx children experience markedly high levels of internalizing symptomatology and these rates appear to continue into middle childhood (Saluja et al., 2004). During adolescence, 35% of Latinx youth (47% girls; 21% boys) report significant feelings of depression and 16% (22% girls; 11% boys) report that they have seriously considered suicide (Centers for Disease Control & Prevention, 2016). Among youth in Texas, home to the second largest Latinx youth population in the USA, research shows that Mexican-origin middle school girls are more than 2 times more likely, and boys 1.6 times more likely, to express suicidal ideation compared with White students, even controlling for a host of sociodemographic and psychological factors (e.g., family structure, discrimination) (Tortolero & Roberts, 2001).

Depression and suicidal ideation are also associated with an array of long-term social (e.g., lower educational attainment, lower income, poor marital quality) and mental health (e.g., anxiety, substance use) problems, including ongoing suicidal ideation and eventual death by suicide (Bridge et al., 2006; Goldman-Mellor et al., 2014; Nrugham et al., 2015; Suominen et al., 2004). In adolescence, one in ten Latinas attempts suicide (Centers for Disease Control & Prevention, 2016), and Latina teens have been shown to reattempt suicide in up to 62% of cases (Hausmann-Stabile et al., 2012), a rate 10 times greater than in other groups (Burns et al., 2008; Goldston et al., 2015).

Current scholarship on pediatric depression and suicidal ideation recognizes the interplay of various (e.g., biological, cognitive, interpersonal) dynamic etiological factors. For example, the role of genetic risk is supported by evidence from family, twin and adoption studies that show a 2- to fourfold increase in the likelihood of major depressive disorder-recurrent unipolar (Lohoff, 2010). Studies of temperament further substantiate the notion of genetic heritability (Compas et al., 2004; Rothbart, 2011). Importantly, though, innate susceptibility is considered a distal factor that influences depression and suicidality via other factors.

Cognitive vulnerability-stress models of depression (e.g., Mezulis et al., 2010) posit that as youth attend to and ruminate on negative stimuli, they experience more stress, feelings of hopelessness, and ultimately depression and/or suicidal ideation (Abramson et al., 1989; Beck et al., 1985; Nolen-Hoeksema, 1991; Nolen-Hoeksema et al., 2008). Disengagement coping strategies, such as withdrawal and avoidance, contribute to social isolation, which exacerbates negative feelings and reduces opportunities for positive reinforcement and support (Manos et al., 2010; Martell, Addis, & Jacobson, 2001). Support from parents and peers acts to enhance positive cognitions and engagement coping skills, whereas conflict or rejection reinforces negative cognitions and disengagement. Family and peer support in this sense is a resource that reduces cognitive vulnerabilities but can also be a positive response to avoidant and maladaptive behaviors that may arise because of an increase in cognitive vulnerability that may arise from a developmental school transition or psychosocial environmental exposures to oppression including discrimination, anti-immigrant sentiment in the media, and overt violence.

For Latinx families, what constitutes support for the individual child or adolescent may vary. Indeed, youth of parents who are harsh and neglectful develop cognitive distortions that increase mental health risk (Ostrander & Herman, 2006; Randolf & Dykman, 1998). For example, a harsh or quick response to maladaptive behaviors may be seen as supportive by some and harmful by others, whereas a delayed response to maladaptive behaviors that is less harsh may be subjectively experienced as neglectful. Several studies have also identified lack of social support as a correlate of suicidal ideation (Mackin et al., 2017; Roberts et al., 1998; Van Orden et al., 2008), but social support is both a cause and consequence of cognitive vulnerabilities and may be especially challenging for bicultural children and adolescents of immigrants negotiating a socioecological environment laden with hostility toward immigrants in general.

Given the host of factors that have been empirically linked to depression and suicidal ideation, scholars now emphasize the need for holistic and integrated models that account for the active, reactive, and interactive nature of youth (Granic & Hollenstein, 2003). Recent efforts to understand the dynamics of cognitive and emotional development have emphasized the role of developing theories using computer modeling and simulation (e.g., Frankenhuis, 2019; Kunnen, 2017; Millner et al., 2020; Yang et al., 2019). Hence, new opportunities exist for understanding the types of dynamically complex interactions of cognitive vulnerabilities associated with depression and suicidal ideation for Latinx children and adolescents.

In this paper, we present a data-driven development of a feedback theory of cognitive vulnerabilities and family support. Specifically, we draw on coding of qualitative key informant interviews to develop a feedback theory that is developed and analyzed (appraisal in the sense of Meehl, 1990) as a formal system dynamics computer simulation model (Richardson, 2011; Sterman, 2018). While the application of system dynamics to understanding developmental trajectories is not new (see Levin & Roberts, 1976) and there have been recent applications to depression (Wittenborn et al., 2015), this paper is unique in drawing on the most recent advances in analyzing loop dominance in developing novel propositions for theory development, appraisal, and testing.

The paper is organized as follows. First, we provide a background on the dynamic nature of psychopathology, the role of school transitions, and the conceptual lens of system dynamics. Next, we describe our approach to developing a data-driven development of a feedback theory using system dynamics. We then describe the specific approach for developing and appraising the theory. This is followed by a discussion about the theoretical implications of the formal feedback model from computer simulation and analysis. We conclude with a discussion on the implications.

## Background

New studies show that up to 20% of young children receiving psychiatric care experience suicidal ideation (Luby et al., 2019; Martin et al., 2016) and that 43% of youth presenting to emergency rooms for suicidality are between the ages of 5 and 10 (Burstein et al., 2019). Pre-adolescent children indicating suicidal ideation are 1.5 times more likely to make a later suicide attempt than those who do not indicate suicidal ideation (Ialongo et al., 2004; Steinhausen & Metzke, 2004). A recent study of pre-adolescents receiving emergency care (for physical and mental health) found that 18% reported suicidality, half of whom engaged in suicidal behaviors *before the age of 10*. Thus, it is critical to understand that suicidal ideation appears to emerge earlier than previously thought, and typically in the context of depression (Cash & Bridge, 2009; Foley et al., 2006; Gibb et al., 2010; Romero, 2014).

Depression is characterized by a constellation of symptoms related to sad or irritable mood, accompanied by behavioral (e.g., sleep changes, anhedonia) and cognitive (e.g., feelings of worthlessness) symptoms. Suicidal ideation (SI) is both a symptom of depression and feature of suicidality (Posner et al., 2007). SI references thinking about, considering, or planning suicide (O'Donnell et al., 2004; Posner et al., 2007; Turecki & Brent, 2016). By the time of adolescence, depression is especially prevalent among girls (Centers for Disease Control & Prevention, 2016; Richardson et al., 2003) along with the expression of SI ranging from passive thoughts about death to actively planning a suicide attempt (Nock et al., 2013; Spirito & Overholser, 2003). One-third of adolescents with SI eventually attempt suicide, with most adolescents with SI attempting suicide (86%) doing so within a year after onset of ideation (Nock et al., 2013).

Importantly, though, depression and SI appear to emerge before adolescence (Dykxhoorn, Hatcher, Roy-Gagnon, & Colman, 2017; Kovacs et al., 2016; Rao & Chen, 2009). Approximately 15% of children under the age of 6 are thought to have clinically significant problems (Centers for Disease Control & Prevention, 2016; Von Klitzing et al., 2015), and the emergence of depression and SI, specifically, has been documented in early childhood (Luby et al., 2016; Martin et al., 2016; Zeanah & Gleason, 2015). According to Luby and colleagues, children as young as 2 to 3 years old experience depressive symptoms, and especially irritability, anhedonia, sleep and appetite changes, and low self-esteem, though these symptoms may manifest more intermittently in early childhood than later in development (Whalen et al., 2015). SI has also been documented in young children, though it may be expressed through drawings or play rather than verbally (Luby et al., 2019).

### The Dynamic Nature of Developmental Psychopathology

Evidence of the dynamic nature of child development comes from researchers interested in cascading constraints*,* or the limited degrees of freedom of behavioral repertoires that narrow the possibilities for a given youth’s developmental trajectory over time (Lewis et al., 1997). For instance, research shows that risk during infancy, such as financial strain or parental psychopathology (e.g., maternal depression), compromises the use of effective parenting skills so that mothers are less likely to create warm, nurturing, and appropriately demanding interactions with their young children. Consequently, children are less likely to develop the self-regulation skills that facilitate a positive transition into school and more likely to exhibit behavior and social problems across settings. These bidirectional links, between parenting and children’s developmental competencies, recur throughout the development to magnify into more serious problems in adolescence (e.g., depression, SI; Dodge et al., 2008). A number of longitudinal studies support these dynamic cascades, in which early disadvantage cumulates into later disadvantage (Eiden et al., 2016). Likewise, studies of depression show the consolidation of depressogenic cognitive styles (i.e., cognitive vulnerabilities) during adolescence, increasing the immediate and longer-term risk for depression by influencing the way in which youth interpret challenges as stressful and overwhelming (Hankin et al., 2009).

### The Transition to School as an Opportunity for Intervention

Transitions occur throughout the development (e.g., birth of a sibling, move to a new neighborhood, family migration). During these transitions, the human ecological system (Bronfenbrenner, 1979) is disrupted or perturbed. The individual and microsystems that were in equilibrium or quasi-equilibrium become disequilibrated, and individual and microsystems that were already in a disequilibrium generally remain in a disequilibrium, albeit a potentially different disequilibrium. Reacting to the perturbation, the individual child must reorganize by drawing upon his or her existing individual and microsystem resources to meet the new challenge. For example, a child moving into a new neighborhood and school from a previously stable set of friends and social expectations must now adapt to the new environment a period of adjustment before reaching a new (quasi)equilibrium with new peers and different social expectations.

We consider depression and SI during transitions into school, a normal yet challenging experience for *all* youth in the USA and one with unique barriers for Latinx youth, as described below. In the US educational system, youth transition into a new level of schooling at three points corresponding with the early childhood, middle childhood, and adolescent stages of development: as they enter elementary school in Kindergarten, middle school, and high school. These transitions are similar in many school systems that require youth to enter an unfamiliar physical setting with new organizational structures, relate to new peers and adults, and master new learning (i.e., academic) challenges (note that public and independent schools that combine elementary with middle school, middle with high school, or provide a seamless K-12 education avoid these transitions). To be successful, youth—regardless of their academic competencies—must have internal and external resources (e.g., self-esteem, teacher support) that can be leveraged to meet the specific challenge of a new school setting (Benner, 2011).

Still, theoretically*,* a child with the right configuration of resources will be able to successfully navigate the transition to school, though resources within the individual (e.g., coping skills) and microsystem (e.g., family support, peer support) will be temporarily unbalanced and disequilibrium before stabilizing with higher-order skills; this represents developmental growth. If, however, the child does not have the resources to meet the challenge, and the demands of the challenge become overwhelming, developmental stagnation or decay may be observed. In other words, when resources are insufficient and/or a poor “fit” for the demands of the challenge, psychopathology may develop (Hendry & Kloep, 2002; Wittenborn et al., 2015). This idea aligns with the diathesis-stress model of depression in which environmental factors—including life transitions—act as stressors that tax the coping capacities of youth to precipitate the onset of depression (Carr, 2008; Hankin & Abela, 2005). What’s more, transitions that have been experienced as stressful earlier in life represent a particular point of vulnerability, suggesting that youth who have had a challenging transition to school earlier in life may be at highest risk for depression and SI during a *subsequent* school transition (Carr, 2008).

A number of studies document an increased risk for adverse outcomes resulting from school transitions, with some estimates suggesting that more than 1 in 3 students experience some type of difficulty (academic, behavioral, social, emotional). Much of this literature focuses on academic performance and school dropout, but some evidence suggests that self-esteem, depression and even suicidality may be impacted as well (Benner & Graham, 2009, 2011; Denner et al., 2019; Gore & Aseltine, 2003; Williams et al., 2017). A study that followed 631 youth from 2nd grade to 8th grade found that the transition from elementary to middle school was associated with increased risk for internalizing symptoms (Nelemans et al., 2018). A separate study with predominately Latinx students found an increase in depressive symptoms after the transition from middle school to high school (Benner, Boyle, et al., 2017; Benner, Thornton, et al., 2017). Indeed, entry into school can be especially stressful for Mexican-origin students and their families (Benner, Boyle, et al., 2017; Benner, Thornton, et al., 2017). Scholars argue that the (dis)continuity, or nonalignment, between home and school environments plays a key role in student adjustment (Reese & Gallimore, 2000; Rogoff, 2003), and this may be especially true during the transition to kindergarten (Rimm-Kaufman & Pianta, 2000). Cultural and language differences intersect with poverty and legal status to further marginalize students. The majority of Latinx youth have at least one foreign-born parent (Child Trends, 2014), and legal status shapes the everyday experiences of children (Abrego, 2016; Dreby, 2012, 2015; Suárez-Orozco et al., 2011). For example, youth of undocumented parents report pressure to avoid detection and fear of authority en route to and while they are at school (Abrego, 2016; Berger Cardoso et al., 2018; Brabeck et al., 2016; Dreby, 2015; Lykes et al., 2013; Rubio-Herhandez, 2015). These stressors can be understood as manifestations of structural racism (powell, 2008). By this we mean, these stressors are not “just” artifacts or events associated with specific cultures or experiences of immigration, but a consequence of an underlying system of structural violence where the exposures to the underlying propensity or risk concentrated in specific populations is the major contributor to morbidity and mortality in a population (Galtung, 1969).

On the other hand, youth and families are resilient, as noted above, and are known to adapt to experiences of marginalization by drawing on culturally derived coping strategies (i.e., “adaptive culture”) that shape development (Garcia Coll et al., 1996). For example, there is evidence that ethnic identity increases during school transitions (French et al., 2006). Also, like all populations, Latinx youth have a wide range of individual resources to draw upon, such as an easy-going temperament, engagement coping skills, high self-esteem, positive relationships with parents, and peer support. Though empirical evidence is limited, past studies document the critical role of support from parents, peers, and teachers in easing the transition to school (Barber & Olsen, 2004; Benner, Boyle, et al., 2017; Benner, Thornton, et al., 2017; Newman et al., 2007; Reis et al., 2009).

### System Dynamics

System dynamics is a theoretical conceptual framework placing the emphasis on understanding the dynamic behavior of a system from an *endogenous or feedback* perspective (Richardson, 2011). By emphasizing an endogenous or feedback perspective, system dynamics draws attention to understanding the reciprocal or cyclic causal effects of systems over time in terms of a set of balancing and reinforcing causal feedback mechanisms. Balancing feedback mechanisms counteract or balance a change or disruption toward a goal. A thermostat in a heating/cooling system is a classic example of a balancing feedback mechanism grounded in the metaphor of servomechanism, but there are many examples of balancing feedback mechanism from the biological mechanisms regulating sleep in the body to information feedback mechanisms of socially correcting learned behavior. Reinforcing feedback mechanisms amplify the direction of initial change. Exponential growth of cellular growth and human reproduction are examples of biological reinforcing mechanisms.

While simple “atomic” patterns of behavior like exponential growth/decline and goal seeking growth/decline can be understood in terms of single feedback loops, more complex patterns involve multiple interacting balancing and reinforcing feedback mechanisms (Ford, 1999). The focus of system dynamics as a conceptual framework and method is on understanding how dynamically complex patterns of system behavior over time are generated by a set of balancing and reinforcing feedback loops. That is, system dynamics is theoretical lens adopted *to see and understand* the dynamics of feedback mechanisms and *complements* the more familiar and traditional acyclic or linear cause–effect assumptions underlying much of our statistical methods.

In system dynamics, the assumptions underlying the mathematical representations are relaxed to include any (piecewise) continuous time system with continuous variables. This includes nonlinear interactions (e.g., X•Y, X/Y) and arbitrary monotonically increasing or decreasing curvilinear functions describing the effects of one or more variables on another. The mathematical implication from this generality in representing feedback systems is that *most representations* are not amenable to analytic solutions typically taught and understood in calculus or ordinary differential equations. That is, there is generally not some way to solve the system of equations analytically to base estimation of parameters confidence intervals. Instead, numerical methods are used to solve the system of differential equations using computer simulation, an approach frequently employed in the basic physical sciences and engineering to develop theory and understand the behavior of complex systems (Palm, 1983).

Where linear cause–effect perspectives may be extended (to a limited degree) to include some aspects of cyclic interactions, system dynamics *begins* with the assumptions of nonlinear feedback interactions over time. Such interactions are central to understanding developmental trajectories in psychology. A child learns from their parent/guardian(s) responses to their own distress and adapts, evolves, or succumbs to behavioral expectations conveyed in the response, which the parent/guardian(s) in turn respond to, and the child learns from this in another iteration that the parent/guardian(s) must respond to, and so it goes—it’s hard to imagine anything more dynamic from a feedback perspective than parenting, and yet it often seems we tend to treat parenting both in practice and research as something that is best served by a linear cause–effect model.

From a clinical perspective, the linear cause–effect system versus nonlinear feedback system has major implications for understanding prevention and intervention in practice. In a linear cause–effect system, the consequences of behavior and actions are attenuated and dampened the more distal the variables are from the causes. Causes that are proximate to the effects are more likely to become the primary sources for explanation and intervention. Hence in suicide prevention among adolescents, research has tended to favor the proximate causes of suicide to the exclusion of more temporally distal causes in early child development. However, in nonlinear feedback systems, the primary drivers are typically feedback mechanisms, and they can be both temporally and causally distant from the effects. The focus of assessment and intervention therefore tends to be on understanding the origins of behavior in terms of the main feedback loops driving the trends in each phase and identifying opportunities for intervention. This can have major implications in practical terms of clinical research and intervention.

For example, whereas research on an intervention from a linear systems perspective may identify associations that increase or decrease a given outcome, clinical interventions based on this may only moderate the effects in the desired direction, but not alter the overall trajectory in some fundamental way. For example, one may find that a given intervention with a family increases the perceived support by the child or adolescent, and that there is a decrease in cognitive vulnerabilities relative to the counterfactual of no intervention, *but still find that the cognitive vulnerabilities are increasing (albeit more slowly) in an absolute sense*. It is important to stress that the clinical research anchored in a linear systems perspective is still beneficial, but a focus on only linear systems to the exclusion of nonlinear feedback systems excludes a priori the very kinds of analysis that may be needed to prevent and alter clinically deteriorating or chronic trajectories into trajectories of recovery. While this may hold for most, the complexity of the interactions increases with marginalization, oppression, and social dilemmas created in immigrant families, Latinx families being one of the most exposed and vulnerable populations in the United States in the context current politics, media, and targeted anti-immigrant sentiment.

Stark ethnic inequalities in depression and SI have long been documented, but extant interventions and their corresponding underlying theoretical models fall short of accounting for the contextual nature of mental health in Latinx youth (Duarte Velez & Bernal, 2007). To date, the Latinx population has been “absent from the field’s clinical trials and research” (Escobar & Gorey, 2018) on depression and suicidality, underscoring the critical need for innovative studies that yield precise, culturally specific targets for the prevention of these negative outcomes (Alegria et al., 2010).

To understand developmental trajectories of Latinx youth better and develop better strategies for prevention and treatment, we need to understand the linear cause–effect interactions *along with* the cyclic or nonlinear feedback dynamics as complementary. This notion of complementarity is akin to the idea of complementarity in quantum physics where to understand the complete reality of an atom, we need to consider both the atom’s behavior as a *particle* and *wave*. That is, in the context of advancing theories in developmental psychology, we need to simultaneously appreciate and incorporate both theories of linear cause–effect mechanisms that underlie many of our mental models and analyses of behavior, *and* the cyclic causes or feedback dynamics. We only get a partial and incomplete view if we favor one over the other.

Many psychology theories in psychology refer to and use arguments grounded in cyclic or feedback effects and across multiple levels from Freud to Bronfenbrenner and Bandura. The notion of feedback is new to neither psychology nor the social sciences (see Richardson, 1991). Nor is the use of ordinary differential equations to represent feedback systems of psychological phenomena (e.g., see Boker & Nesselroade, 2002; Levine & Fitzgerald, 1992). What is new are the tools we have to develop and analyze/appraise theories using computer modeling and simulation.

Specifically, new methods are available and implemented in standard system dynamics modeling software for analyzing the influence of feedback mechanisms on the dynamic behavior of systems in terms dominant feedback loops (for an overview of this development, see Ford, 1999; Hayward & Boswell, 2014; Hayward & Roach, 2019; Oliva, 2016; Richardson, 1995; Sato, 2016; and Schoenberg et al., 2020). In particular, the recent developments implemented in isee Systems Stella Architect (2020) using loop scores (Schoenberg et al., 2020) provide a computationally efficient way to analyze and visualize feedback systems in terms of the relative contribution of each feedback loop on the overall dynamics of a system.

Normalized loop scores compute the relative contribution of each feedback loop to the dynamics of a system at a given point in time. The scores are normalized on a − 100 to 100 percent scale with negative values reflecting balancing behavior or loops, and positive values representing reinforcing behavior or loops. The contributions of all the loops are then summed and the resulting value corresponds the overall behavior of the system at that point in time. The resulting analysis allows one to identify the dominant loop sets (i.e., the set of most influential loops) at any given point in time.

The clinical significance of identifying dominant loop sets is arguably still theoretical, but it opens entirely new avenues of inquiry for developing nonlinear feedback theories of developmental trajectories along with a means to analyze the theories to novel prevention and intervention strategies. For example, clinical interventions targeting the dominant loops are theoretically able to alter a trajectory of psychopathology toward healing and recovery, whereas interventions targeting non-dominant loops will not alter the trajectory even though the intervention might attenuate the severity of a condition.

## Approach

The transition into school represents a natural developmental challenge during which preventive interventions may interrupt the emergence of depression and SI in Latinx youth. Thus, the present study uses principles of system dynamics modeling (Forrester, 1990; Richardson & Pugh, 1986; Sterman, 2000) based on qualitative interview data to develop a formal feedback theory of risk for depression and SI among Latina youth during school transitions.

Computer simulation is used to solve the system of differential equations through numerical integration (Burden & Faires, 1989; Palm, 1983) where the solution is the simulated behavior of the system over time. Although the models can be developed and simulated using any general purpose programming language, specialized software packages specific to system dynamics make the job much easier (e.g., isee System’s Stella Architect, Ventana’s Vensim, Powersim Studio). These packages provide a graphical toolkit for creating the model and specifying the general form of the differential equations along with standard libraries of functions commonly used and features for specifying and checking the dimensional consistency of a model. Some packages such as Stella Architect also support the creation of online interfaces that be deployed on websites such as exchange.iseesystems.com. This allows audiences to experiment with the simulation models on their own and develop insights into the relationships between structure and behavior.

The process of developing a feedback theory and translating the theory from a diagram into a formal model that can be simulated on a computer is highly iterative. A modular approach is taken to build and test each component of the system before moving onto formulating the next component (Homer, 2012). The equations typically involve parameter values (e.g., proportions, doubling times, half-lives, time constants, hazard rates, hazard rate ratios) and values for initial conditions at the start of the simulation for all stock or state variables. These are initially assigned provisional values as “priors” and then varied over their logical ranges as each equation is tested to establish its logical consistency. As modeling progresses, the priors can be updated with better estimates from secondary data analyses, systematic reviews or meta-analyses, and primary data including key informant interviews, focus groups, and expert panels.

For example, one might have an equation that involves the proportion of people developing clinical signs of depression over a year, which can logically vary from 0 to 1. While the actual proportion for a given population should be used and can be based on existing data, systematic reviews, etc., the expectation in system dynamics is that the model is able to generate logically plausible results over the *entire logical range* of values. That is, one is building a model of a theory that can represent both the current state of the real system *and* cover a wide range of alternative worlds.

A common result from this type of iterative model building and testing is that the dynamic behavior of a system is often *insensitive* to assumptions about the numerical values of parameters and initial conditions, at least in the qualitative sense of the overall trends. That is, one can often vary the values by as ± 50% or more and see the same general pattern of dynamic behavior for a system. Moreover, one also finds *some* parameters and initial conditions where small changes can significantly alter the dynamics, e.g., changing the behavior from a pattern of exponential growth to oscillator, emergence of tipping points, and other phenomena associated with dynamical systems. Hence, the priority in terms of finding solid empirical estimates for parameter values and initial conditions tends to be on the values that appear to qualitatively alter the behavior patterns.

When the simulation model can reproduce the behavior pattern(s) of interest, one has shown that the theory *can* generate the behavior patterns. That is, one has demonstrated via computer simulation that the formalized theory is a logically consistent explanation for the phenomenon of interest. While behavior reproduction of this sort is a relatively weak test, many verbal theories tend to initially fail even this test when formalized and simulated on a computer. The consequence of empirically testing a hypothesis grounded in a logically inconsistent theory is an inconclusive result regardless of the level of sophistication in a statistical test (Meehl, 1990). What formal theory specification and computer simulation therefore provides is a faster and less expensive way to conduct theory appraisal and discover novel hypotheses that can be empirically tested (Kunnen, 2017; Millner et al., 2020). This becomes especially important when considering complex nonlinear and multilevel interactions of feedback mechanisms over time where even relatively simple feedback systems can be counterintuitive.

Central to system dynamics is explaining the dynamics of a system in terms of the specific underlying feedback mechanisms. The notion that one or more feedback loops are determining the qualitative pattern of behavior is referred to as loop dominance (Richardson, 1995). While there is a mathematical relationship between the parameter values for a specific causal link and the relative strength or influence of a feedback loop, the very nature of a feedback loop and the behaviors that arise from multiple feedback interacting over time results in shifts in loop dominance. This is a mathematical consequence of relaxing assumptions about linear cause–effect latent causal structures to include nonlinear feedback mechanisms. Existing statistical methods such as multilevel modeling and structural equation modeling can be used to establish general associations between parameters and trends over time (e.g., growth curve modeling) and there are some means to represent non-recursive relationships; however, these methods tend to break down when systems involve shifts in feedback loop dominance (Hovmand & Chalise, 2015). System dynamics provides an explicit approach to specifying and analyzing the feedback mechanisms that consider shifts in feedback loop dominance.

The substantive implication of identifying dominant feedback loops is that they identify feedback mechanisms that can be intervention targets for changing *the dynamics* of a system. Put differently, there are many places in a system where one can intervene and interventions with measurable effects will change the numeric values, but if the target is not a dominant loop, it will not alter the fundamental pattern of behavior. For example, if cognitive vulnerabilities are increasing exponentially, intervening on the dominant loop will alter the pattern (e.g., to a stabilizing pattern of logistic growth or decline) while intervening on a non-dominant loop will only moderate the pattern of exponential growth. It is important to stress that this does not mean that intervening on a non-dominant loop isn’t helpful (reducing the level of anxiety, even if it is continuing to escalate is still better than doing nothing or making it worse). However, for complex dynamical phenomena that may be difficult to prevent or treat, being able to fundamentally change the dynamic pattern is critical.

In this paper, we present the Developmental Transitions (version 2–2-9) system dynamics model as a formal representation of a development of a feedback theory of cognitive vulnerabilities and family support. We focus primarily on the development and analysis of the system dynamics model and only provide a summary of the qualitative methods.

## Data

Data supporting the model came from a secondary analysis of qualitative interviews exploring suicidal behaviors among adolescent in low-income families in New York City. Participants in the qualitative phase of the larger study included 73 Latinas aged 11 to 19 who had self-harmed within six months prior to the interview and 66 Latina adolescents with no reported lifetime history of self-harm. IRB approval was granted at all institutions involved in project activities, and each participant (adolescents and their caregivers) provided assent and consent to participate in the study.

In this paper, we draw on a subsample of participants. Participants with histories of self-harm were selected for analysis if they explicitly stated suicidal intent in the qualitative interview (*n* = 37). Participants with suicidal intent were then matched to adolescents with no histories of self-harm based on age and place of birth, resulting in a final subsample of 60 participants (30 who had attempted suicide, and 30 with no reported history of suicidal behaviors, and 7 were dropped from the analysis because they could not be matched). Over 60% of the participants in this subsample were also matched by legal status and Hispanic subgroup. In the total subsample, seven Hispanic subgroups are represented: Colombian, Dominican, Ecuadoran, Mexican, Puerto Rican, Salvadoran, and Venezuelan. Approximately 40% of participants were born outside the United States. The average age of participants was just under 16 years.

All adolescents participated in an in-depth qualitative interview. Each participant with a reported history of suicidal behavior was guided through a detailed, retrospective account of her suicide attempt to elicit the psychological and social dimensions of the experience prior, during, and after the attempt. All participants were asked about the dynamics of family life (e.g., family relationships, conflict management, roles and responsibilities, discipline); perspectives on being an adolescent (versus child or adult); systems of social support; school and extracurricular activities; future aspirations and goals; and sociocultural experiences (e.g., the meanings of being a young Latina). Interviews were conducted by bilingual Latinas with clinical licenses in social work or psychology, and all interviewers were trained in qualitative methods to facilitate the collection of specific, detailed narratives. Interviews were conducted in either Spanish or English, depending on the preferences of participants. Qualitative interviews ranged from 25 to 70 min in length and were digitally recorded. Interviews were transcribed and analyzed in the language of the interview by a team of bilingual researchers.

## Causal Mapping

To develop a feedback theory, we drew on analytical techniques established in grounded theory (Strauss & Corbin, 1998). A grounded theory approach has been well established in system dynamics research and shown to be consistent with the strategies needed to develop a feedback theory (for an overview, see Kim & Andersen, 2012). Analysis proceeded in three stages: (1) open coding, (2) axial coding, and (3) selective coding. A team-based approach was used to integrate the expertise of the research team, protect against bias, and encourage multiple perspectives in coding and analysis (Ryan & Bernard, 2000). Details of the approach used to go from qualitative interviews and analysis to causal loop diagrams are described in a separate paper. The basic approach codes variables as causes or effects and then determines whether the relationship in that part of the text indicates that the cause increases or decreases the effect. Table 1 summarizes the variables and causal structures from the qualitative interviews and analysis along with supporting quotes.

**Table 1:** Example of causal structures identified in qualitative analysis (bold text in parentheses indicates coded variables)

Cause	Effect	Relationship	Participant quote
(Lack of) Family Support	Cognitive Vulnerabilities	Increases	“I was angry because my mom was screaming at me. And she was like making me feel like everything was my fault (**lack of family support**). I felt like no one understanded [sic] me. Like the way I am, who I am. I felt like my dad would not love me anymore. And that my mom probably hated me (**cognitive vulnerabilities**).”
Cognitive Vulnerabilities	Avoidant Coping	Increases	“I got all these feelings in me (**cognitive vulnerabilities**). And for me, I can’t tell nobody ‘cause they might say something. I can’t really tell my mother (**avoidant coping**). So for me, I just get crazy [with] all this stuff that is going on to me (**cognitive vulnerabilities**). I keep it to myself (**avoidant coping**). So they build up, until I can’t anymore.”
Avoidant Coping	Maladaptive Behaviors	Increases	“During the day I was, I was trying to hide my feelings. I was feeling very, very depressed. And I was trying to hide my feelings (**avoidant coping**). I tried as hard as I could to keep on with my act. So that’s when I started thinking, ‘Oh, I should take something to numb my pain.’ Nothing mattered to me. Nobody mattered to me. So, I started to use marijuana with cocaine (**maladaptive behaviors**).”
Maladaptive Behaviors / Avoidant Coping	Family Support	Increases	“Like my freshmen year, I messed up horribly. Like I used to cut school a lot and started partying (**maladaptive behaviors**). I’d go home and do nothing (**avoidant coping**). My mom, she started getting clues and started noticing. She’d always tell me “You can always come and tell me,” (**family support**). And ever since then our relationship has opened a lot.”

The resulting coding was used to build a causal loop diagram representing the feedback theory underlying the dynamics. It is important to stress that a feedback theory emerged by synthesizing the coding from multiple interviews, not from a single individual. That is, the resulting feedback theory was not emerging from multiple individuals reporting the same links creating similar feedback theory, but from aggregating individually reported links to form a feedback theory.

This introduces a limitation with respect to the generalizability of the feedback theory, but it is important to remember that the focus in this paper is on developing a data-driven feedback theory that is sensitive to the dynamic complexities of Latinx children and youth. Our primary goal is to develop a feedback theory relating cognitive vulnerabilities and family support that is sensitive (biased) toward picking up on the dynamics faced within Latinx families. Before we can ask whether a given feedback theory is generalizable, we first need to have a feedback theory, and we argue such a theory is better served by being grounded in data from individuals with the relevant lived experience aggregated than an abstract alternative. Once we have a feedback theory and understand the implications through a formal theory, i.e., appraisal by formulating the model and simulating the dynamics, we are in a much better position to rigorously test and refine a feedback theory empirically using qualitative and quantitative methods.

## Model Formulation

The model was developed using Stella Architect (2.2.1). Stella Architect (isee Systems, 2020) is a commercially available software package for developing, simulating, and analyzing mathematical models of nonlinear feedback systems as a system of coupled ordinary differential equations. Stella Architect uses numerical methods for solving a system of ordinary differential equations over time (e.g., Euler integration, Runge–Kutta integration). While these models can in principle be developed and simulated using any software package that can numerically solve systems of ordinary differential equations (e.g., MATLAB, Mathematica, deSolve or ODE packages for R), Stella Architect provides additional features such as dimensional consistency tests, publishing models online via isee Exchange, and loop dominance analysis using loop scores.

This is an individual level model representing the dynamics of cognitive vulnerabilities and family support from 0 to 21 years of age. Formulation of equations followed standard conventions of system dynamics (e.g., Ford, 1999; Forrester, 1990; Richardson & Pugh, 1986; Sterman, 2000) with all units specified and equations dimensionally consistent. Variables were quantified on a 0–100 ratio scale with provisional units assigned to psychological variables consistent with principles of system dynamics (e.g., “FS Units” for Family Support).

Although there has been some debate about the appropriateness of assigning what may seem arbitrary units to intangible variables, in particular, Jacobsen and Bronson (1987) arguing that a dimensionless quantity such a percentage or proportion is a better choice, specifying units provides an important check that equations are being formulated in a consistent manner. Moreover, once formal model of a feedback theory has been developed, one can identify and develop measurement scales with appropriate indicators latent constructs paying particular attention to the fact that variables in system dynamics models are continuous and at the ratio level of measurement (Levine & Lodwick, 1992).

Developmental transitions are represented as pulse functions that occur at school transitions (ages 5, 11, 14, and 18) with a magnitude of 50 percent (on a 0 to 100 scale) reflecting a moderate shock or increase in cognitive vulnerabilities. We then explored the model and possible behavior patterns by manually adjusting the parameter values and initial conditions (using Stella Live) over logical ranges to see whether the feedback theory could generate a diverse set of individual trajectories over time. In particular, we want to be able to replicate a variety of representative trajectories from a child who experiences a developmental shock from the school transitions but recovers quickly with family support to a child who is already predisposed and experiencing an increase in cognitive vulnerabilities. The initial conditions and parameter values were then saved to create a set of hypothetical cases representing different combinations of dynamics between cognitive vulnerabilities and family support.

An online interface was developed using Stella Architect and available at https://tinyurl.com/yr9r3md2. This allows a user to explore the six scenarios in addition to changing parameters and assumptions. For example, although we assume linear (proportional effects), we represented these relationships using table functions that can be modified by the user to see the implications of relaxing this assumption.

## Loop Dominance

Over the years, there have been a variety of techniques for assessing loop dominance ranging from informal experiments where one deactivates a loop to more formal techniques, but until recently, these have largely required modifying the model as the techniques were not included as part of standard software packages. However, with the introduction of loop scores in Stella Architect in 2020 (Schoenberg et al., 2020), it is now possible to routinely visualize and study the shifts in loop dominance.

The notion of loop scores is especially attractive due to its similarity to interpreting path scores in path analysis and structural equation modeling. Essentially, loop scores are calculated as the product of link scores in a feedback loop, where the link score is calculated as the proportion of the overall change in a variable over time, *X(t) – X(t-dt)*, that can be attributed to the change in the antecedent or causal variable in the link while holding other variables constant. For details on the algorithm for identifying the feedback loops and calculating the loop scores, see Schoenberg et al. (2020).

Positive loop scores indicate a reinforcing loop while negative loop scores indicate a balancing loop. The net effect of all the loops at any point in time can then be calculated by summing the loop scores. If the sum is greater than 0, the reinforcing loops are driving the overall trend in the system. If the sum is less than 0, the balancing loops are driving the overall trend in the system. And if the sum equals 0, the system is in a dynamic equilibrium.

Loop scores are typically normalized by summing the absolute value of all the loop scores and then scaling the scores to -100 to 100 percent, thereby providing an easy interpretation of how much any given loop or set of loops is contributing to the overall dynamics of a system. A single loop can be dominating the behavior of a system when its loop score is more than 50%, but one can also have situations where there are no single loops dominating the behavior of a system during a particular behavior phase. In these situations, there are a set of feedback loops where the sum of the absolute value of their loop scores is greater than or equal to 50%. To visualize the dynamics of the loop scores, we added variables for each of the main feedback loops in Stella Architect.

## Simulation

To generate the results in a replicable document, we repeated the simulation, analysis, and plots in RStudio using Stella Simulator 2.2.1 as an R Markdown document. Stella Simulator is a version of Stella Architect without a graphical user interface (GUI) suitable for computationally intensive simulation and analysis. This was more for conveyance as Stella Architect also supports running simulations through the system command line, but each simulation run opens and closes the GUI and hence tends to be significantly slower.

## Documentation

The full equation listings with documented equations are included as an appendix along with the R Markdown document for running the simulations following standard conventions for reporting system dynamics modeling results (Rahmandad & Sterman, 2012). The Stella Architect file along with all analysis is available as a public GitHub repository at https://github.com/CBSDLab/Developmental_Transitions consistent with best practices for computational modeling.

## Theoretical Implications

This section reports the theoretical implications from the casual mapping based on the qualitative interviews and analysis and development of the formal simulation model. Development of a formal simulation model often leads to identifying additional causal links or structures that need to be included for the feedback theory to be a complete system dynamics model.

### Revised Feedback Theory

**Fig. 1:**
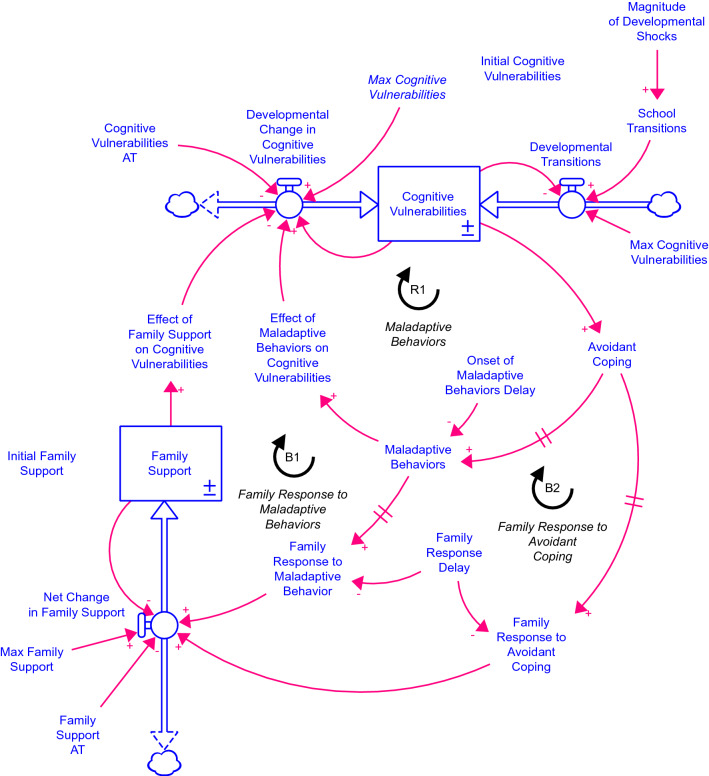
Stock and flow representation for the formal model of the feedback theory

The complete system dynamics simulation model is shown in Fig. 1. Boxes represent accumulations or stocks (state variables). Double lines with “valves” represent flows or rates of change. Clouds represent “sources” or “sinks,” i.e., material and information boundaries of a system. Links with arrows represent causal links between auxiliary variables (also called converters) and links with double lines across represent a delay or lagged effect. Plus ( +) signs indicate a positive association between cause and effect while negative ( −) signs indicate a negative association between cause and effect. Major loops are labeled with an ‘R’ prefix are reinforcing while loops with a ‘B’ prefix are balancing.

There are two main stocks or state variables (*Cognitive Vulnerabilities* and *Family Support*), which each has an initial value (i.e., *Initial Cognitive Vulnerabilities* and *Initial Family Support*) that corresponds to the level of the stock at age 0 and ranges from 0 to 100. There are also three delays modeled as first-order information delays or smooth functions. They represent the lagged effect of one variable on another. For example, there is a delay between changes in *Avoidant Coping* and changes in *Maladaptive Behaviors*. The average length of this delay is determined by the constant parameter *Onset of Maladaptive Behaviors Delay*. The model also assumes that there is a delay in the family recognizing changes in *Avoidant Coping* and *Maladaptive Behaviors*, which is determined by the parameter *Family Response Delay*.

The model has three major feedback mechanisms (R1, B1, and B2 in Fig. 1). First, an increase in *Cognitive Vulnerabilities* leads to an increase in *Avoidant Coping*, which contributes to a delayed onset and increase in *Maladaptive Behaviors*. The increase in *Maladaptive Behaviors* then increases the *Effect of Maladaptive Behaviors on Cognitive Vulnerabilities*, which then “feeds back” to further increase the *Developmental Change in Cognitive Vulnerabilities* forming the reinforcing feedback mechanisms of maladaptive behaviors (R1 in Fig. 1).

As a reinforcing feedback loop, maladaptive behaviors (R1) can form a “vicious” or “virtuous” cycle. For example, during an initial increase in *Cognitive Vulnerabilities* due to developmental transitions, the increase is reinforced by R1 to increase *Cognitive Vulnerabilities*, leading to even more *Avoidant Coping* and *Maladaptive Behaviors*, and thus forming a vicious cycle. However, the same feedback mechanism can also work as a “virtuous cycle.” As *Cognitive Vulnerabilities* decline (e.g., with family support and/or natural recovery), *Avoidant Coping* decreases leading to less *Maladaptive Behavior* which then lessens the *Effect of Maladaptive Behaviors on Cognitive Vulnerabilities,* reducing the *Cognitive Vulnerabilities* even more, and hence now forming a virtuous cycle.

How fast *Cognitive Vulnerabilities* change is an individual trait represented by *Cognitive Vulnerabilities AT,* a time constant. Longer time constants or adjustment times (AT) slow the responsiveness to changes in the effects of maladaptive behavior and family support. We assume that the adjustment time is the same for both effects as this would be an individual trait of the child and that this is constant over time. Both assumptions could be relaxed to explore their theoretical implications on the dynamics of cognitive vulnerabilities and family support.

There are two balancing mechanisms that respond to increases in *Cognitive Vulnerabilities*: family response to maladaptive behaviors (B1 in Fig. 1) and family response to avoidant coping (B2 in Fig. 1). First, an increase in *Cognitive Vulnerabilities* that leads to an *Increase in Maladaptive Behaviors* also leads to an increase in *Family Response to Maladaptive Behaviors*. This represents the family’s recognition that the child or adolescence is engaging in maladaptive behaviors. There is a delay between changes in maladaptive behaviors and family recognition represented by the *Family Response Delay*. When maladaptive behaviors are increasing, this means that the family’s recognition of maladaptive behaviors will be lagging and hence underestimating the level of maladaptive behaviors. For example, families may be in unaware of the maladaptive behaviors, reports from schools may be delayed, and families may deny or minimize the severity of the behavior. However, this same delay also affects the family’s perception of when maladaptive behaviors are declining. In this situation, the family perceives maladaptive behaviors to be higher than the actual level of maladaptive behavior. For example, family perceptions may be based on what happened in the past without considering more recent changes.

In both cases, the level of *Family Response to Maladaptive Behavior* drives the level of *Family Support*. The models assume that *Family Support* is always in the beneficial direction (a strong assumption that can be relaxed for further exploration). It takes time to mobilize and adjust family support in response to changes in maladaptive behaviors, which is represented by the *Family Support AT* or adjustment time. Longer family support adjustment times means that the family is slower to mobilize to increase *Family Support* in response to a recognized increase in maladaptive behaviors, and slower to stepdown family support as maladaptive behaviors decline. Increases in *Family Support* increase the *Effect of Family Support on Cognitive Vulnerabilities*, which feeds back to lessen or “drain” the stock of *Cognitive Vulnerabilities*, forming a balancing feedback loop (B1 in Fig. 1).

Second, with an increase in *Cognitive Vulnerabilities* that leads to an increase in *Avoidant Coping*, there is a *Family Response to Avoidant Coping*. Like the family response to maladaptive behaviors, this represents the family’s recognition of the child’s avoidant coping behavior and is delayed. The length of the delay, *Family Response Delay*, is assumed to be the same as the delay in the family’s response to maladaptive behaviors. Again, such an assumption can be relaxed in future studies to consider different styles of family responses to maladaptive and avoidant coping behaviors in children. The increase in *Family Response to Avoidant Coping* then leads to an increase in *Family Support*, which then feeds back to decrease or “drain” the stock of *Cognitive Vulnerabilities* forming a second balancing feedback mechanism (B2 in Fig. 1).

There are additional elements shown in Fig. 1 needed to formulate a complete system dynamics model. These include minor feedback loops formed when adding a link from a stock to a flow used in common formulations of equations and maximum values of stock that limit the range. Some variables appear twice in Fig. 1 (e.g., *Max Cognitive Vulnerabilities*), but this is merely done to improve the layout of the diagram and does not represent two separate variables (variables in italics in Fig. 1 represent another instance or “shadow” variable).

### Simulation Analysis

**Fig. 2:**
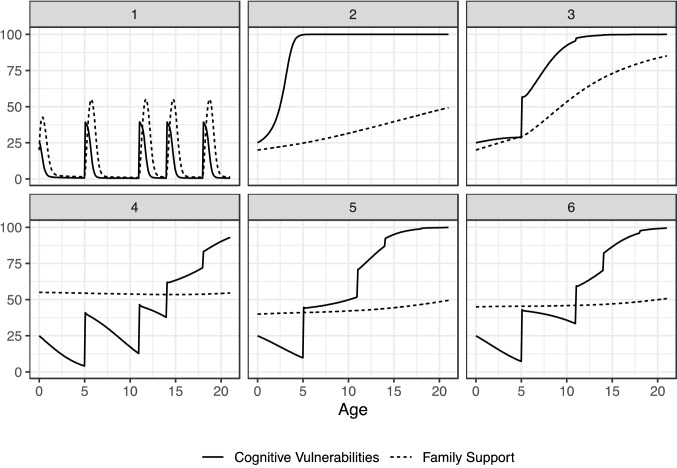
Baserun computer simulations for hypothetical individual (*N* = 6) dynamic patterns of *Cognitive Vulnerabilities* and *Family Support* by developmental age

Figure 2 shows the dynamics of cognitive vulnerabilities and family support for six different hypothetical individuals in response to a series of developmental transitions. Case 1 illustrates a child with a series of transitions with a rise in cognitive vulnerabilities followed by a rise in family support, which then leads to a relatively quick reduction in cognitive vulnerabilities and recovery. Case 2 shows a child predisposed to develop cognitive vulnerabilities which escalate and eventually reach the maximum level of 100 with the family support increasing with age, but without any effect on recovery. Case 3 shows the trajectory of a child where cognitive vulnerabilities appear to be stabilizing with the increase in family support only to increase with the transition into school. Family support increases in response but the cognitive vulnerabilities continue to escalate and become chronic without recovery.

Case 4 shows an individual where the child is recovering for the first two school transitions and has high family support, but recovery is only partial with respect to the pre-transition level of cognitive vulnerabilities and the cumulative effect is that by the third school transition, the child crosses a tipping point and cognitive vulnerabilities escalate and continue to increase into late adolescence. Cases 5 and 6 show a similar pattern to Case 4 with the difference being when the tipping points are crossed. It is important to note that the tipping points are caused by the behavior of the interacting feedback loops and not because of some predetermined threshold function.

**Fig. 3:**
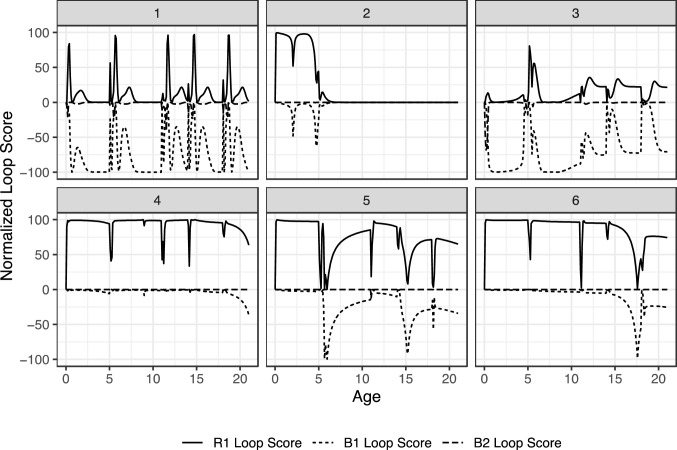
Corresponding loop scores for the three main feedback mechanisms (R1, B2, and B2)

Figure 3 shows the corresponding results for the loop scores for each hypothetical case. Recall that the loop scores are normalized so sum to 100% and that positive values reflect reinforcing loop behavior while negative values indicate balancing loop behavior. The loop labels in Fig. 2 (R1, B2, and B2) correspond to the loops described earlier in Fig. 1.

Each of the cases in Fig. 3 depict periods of relative stability in loop scores interrupted by brief periods of instability. Notable is the fact that the frequency of the dynamics of the loop scores during these periods of instability are much higher than what would be directly observed in the dynamics of *Cognitive Vulnerabilities* and *Family Support.* Initially, we suspected this may have been a numerical artifact from the use of table functions or the use of a discrete pulse function to represent the school transitions. To check this, we (a) built and tested a version of the model that used algebraic expressions for the table functions that were continuously differentiable and (b) another version that smoothed the pulse function with a first-order material delay reasoning that the developmental shock is not instantaneous but distributed over time. Neither of these modifications changed the patterns shown in Fig. 2 significantly.

Through more careful analysis of the model and by changing the initial conditions and parameters continuously, it became apparent that the timings of these transitions were shifting continuously with the parameters. That is, adjusting the parameters and initial conditions changed the placement of the shifts in loop dominance, but not the patterns themselves. If the oscillations in the loop scores were a numerical error, we would expect the patterns to shift in discrete jumps from one pattern to another. Hence, we interpret the patterns shown in Fig. 3 to be results of the underlying dynamics of loop dominance patterns.

## Implications and Conclusions

Depression and SI are prevalent and costly public health problems that contribute to poor long-term health and productivity and increased risk of mortality. Stark ethnic inequalities in depression and suicidality have long been documented, but extant interventions and their corresponding underlying theoretical models fall short of accounting for the contextual nature of mental health in Latina youth. The present study builds on secondary analysis of qualitative interviews to develop a formal feedback theory of risk for depression and SI among Latina youth during school transitions. We capitalize on recent advances in systems science to apply system dynamics modeling, focusing on several well-established correlates of depression and SI: cognitive vulnerabilities, family support, avoidant coping, and maladaptive behaviors. The results represent several contributions toward advancing our scientific understanding of the dynamics of cognitive vulnerabilities and family support among Latinx children and adolescents and potential applications for developing novel approaches to screening and prevention of depression and suicide.

First and foremost, the fact that a relatively simple feedback theory involving two state variables or stocks and three interacting feedback mechanisms can generate a diverse representative pattern of trajectories illustrates the potential power of drawing on system dynamics to develop parsimonious theories that can account for high level of dynamic complexity. Second, that the feedback theories can be developed from qualitative interviews using grounded theory illustrates the feasibility of developing highly contextualized accounts about interactions in human development over time (age). For example, this study used a secondary analysis of qualitative data from a prior study to develop a novel feedback theory. Hence the opportunities to draw on existing qualitative studies as well as design and collect new data to support a grounded theory approach is realistic. It is important to note, however, that while the feedback theory applies to developmental trajectories for children and adolescents, the sample of interviews was limited by both the ages of participants and the specific time of the interviews. That is, we did not follow individuals longitudinally nor did we draw on data from multiple age cohorts within the same study. Both would be limitations if one were trying to go directly from generalizing empirical findings from a relatively small set of interviews conducted at one point of time to a larger population across both developmental and historical time. However, that is not the main aim of this paper, which is to develop a data-driven feedback theory of cognitive vulnerabilities and family support. This form of theory development, we have argued, is necessary to advance novel propositions that can be tested in subsequent empirical research.

Third, the discovery of a rich underlying dynamics in the loop scores suggests novel directions for future research designs. From prior field work using group model building (Hovmand, 2014), we have often anecdotally observed individuals to be able to recognize and identify with specific feedback mechanisms as they experienced them. That is, individuals in community-based workshops have often spontaneously pointed to a feedback mechanism and recounted a story or period in their lives when they experienced that feedback mechanism. This suggests that people may be aware of specific underlying dynamics reflected by the loop dominance dynamics and hence able to report on their experience in semi-structured qualitative interviews or survey instruments about their perception of these dynamics. Hence, this opens a new way to more rigorously develop and test feedback theories of human development.

More specifically, the mathematics of feedback systems is such that the models are inherently underdetermined requiring additional work to rule out equivalent mathematical representations. It is important to note that this issue is not limited to system dynamics, but characteristic of more complex latent causal structures in multivariate analyses (Bollen, 1989). Generally, the issue is that there can be any number of mathematical representations that can generate the same patterns of behavior. Hence finding good statistical fit has a limited value until one has been able to rule out the equivalent models leading to a weaker theory.

However, the dynamics of loop scores provide a much sharper way of testing a feedback theory, going beyond replicating a known pattern to proposing specific underlying behaviors that follow directly from the feedback theory, but would be a priori unknown to the researcher. As such, the dynamics of the loop dominance as operationalized by loop scores present novel hypotheses that can be empirically tested. This ends up being a much stronger theoretical claim than what Meehl (1990) was initially proposing in arguing for more theory specification and appraisal. And, that we may be able to do this through qualitative or quantitative methods is interesting enough to warrant further study in developing new methods. The significance of this would be a major methodological breakthrough in how we go about understanding the dynamics of human development.

Future studies can build on the explicit formulation of feedback theory along with the implications from simulations and analysis to design and test specific propositions to revise or replace the feedback theory. For example, a future study could be designed to conduct and analyze qualitative interviews across a more varied set of age cohorts to see whether the feedback theory can account for the developmental trajectories described by interviewees. The contribution in having a formal simulation model of the feedback theory is that one can both design questions to probe for confirming/disconfirming data more efficiently in interviews and answer whether a novel finding from the interviews was covered by the feedback theory by conducting the computer simulation (i.e., parameterizing the model to the conditions of the interviews and then seeing whether the dynamics are consistent with what interviewees described). Another possibility is taking advantage that schools and school systems differ in their exposure to developmental transitions vis-à-vis school transitions. For example, while many public-school systems are organized into elementary, middle, and high school transitions, many independent and some public-school systems are not. This provides a basis for a study to test the feedback by comparing two populations in a matched comparison or propensity score design.

Fourth, the system dynamics model can be used to conceptualize and develop novel screening and prevention strategies. Although the model was designed as a “proof of concept model,” it generates a sufficient range of dynamics of individual trajectories that can be used to generate a synthetic data set of a population (e.g., school district) and conceptually test various approaches to screening and prevention in a simulation study. Of particular interest to developing better depression and suicide prevention strategies for Latinx children and youth is finding ways to identify individuals who may be at higher risk over the long term based on their response to the development shock of a school transition. Using a simulation model for this type of preliminary work is particularly valuable when considering methods such as a machine learning because one can systematically develop the algorithms with no measurement error and bias, and then gradually relax these assumptions to see how performance of an approach degrades with measurement error and bias. This may lead to either identifying profiles based on already observed data or suggest directions for developing novel screening tools.

In developing a simulation model, we made several strong simplifying assumptions. For example, we often assumed that the adjustments times were symmetric with respect to the direction of change or the same for what might be two distinct processes. We also assumed that family support was always in the beneficial direction. And we largely ignored the fact that families often have more than one child and parents are learning about parenting from experience and a variety of information sources while facing their own expectations and pressures. Relaxing these assumptions would lead to a more realistic model and should be considered in future extensions and studies, but they are unlikely to detract from the main results that a relatively simple feedback model can generate a significant amount of dynamic complexity.

In doing so, we provided a formal feedback theory involving continuous measurements, but we did not address the measurement problem of shared method variance in or common method bias in measurement (Podsakoff et al, 2003). That is, we developed a dynamic feedback theory and represented this theory in a formal mathematical model that can be simulated and analyzed using computer simulation, but as a continuous time and continuous variable model, we never specified how one might go about measuring these constructs as such. We have argued that identifying the dominant loops via loop scores provides an avenue into future research in qualitative interviews and quantitative analyses for discriminating between what may seem to be equivalent models, but in doing so, still have not answered the question about how one might go about collecting these data to avoid the problem of shared method variance associated with self-reported measures.

How do we advance knowledge in developing more effective assessment and intervention strategies for preventing suicide, especially among our more vulnerable and exposed populations? We do this by using methods that can incorporate and reflect the dynamic complexities faced by children, adolescents, and their families across the lifespan. System dynamics is one approach.

In this paper, we focused on understanding the dynamics of cognitive vulnerabilities and family support among Latinx children and adolescents by developing a formal feedback theory and computer simulation model. This work represents a true mixed-method approach combining qualitative grounded theory of key informant interviews with computer simulation of a quantified formal feedback theory and analysis to generate novel hypotheses that can be empirically tested. Of relevance to the topic, the paper highlights how even relatively simple interactions between an individual child, their family, and the school as their social environment can generate complex dynamics that might otherwise be difficult if not impossible to organize using more traditional statistical models. Advances in depression suicide prevention across the lifespan need to consider these complex interactions if we are going to be able to make a significant impact on reducing the disparities of depression, suicidal ideation, and attempts among Latinx youth.

## Supplementary Information

Below is the link to the electronic supplementary material.

Supplementary file1 (DOCX 26 kb)
