# Correction to: System Dynamics of Cognitive Vulnerabilities and Family Support Among Latina Children and Adolescents

**DOI:** 10.1007/s10567-022-00397-1

**Published:** 2022-03-18

**Authors:** Peter S. Hovmand, Esther J. Calzada, Lauren E. Gulbas, Su Yeong Kim, Saras Chung, Jill Kuhlberg, Carolina Hausmann-Stabile, Luis H. Zayas

**Affiliations:** 1 School of Medicine, Case Western Reserve University, Cleveland, USA; 2 Steve Hicks School of Social Work, The University of Texas, Austin, USA; 3 Department of Human Ecology, The University of Texas, Austin, USA; 4 SKIP, Washington University, St. Louis, USA; 5 System Stars, LLC, St. Louis, USA; 6 Graduate School of Social Work and Social Research, Bryn Mawr College, Bryn Mawr, USA

## Correction to: Clinical Child and Family Psychology Review 10.1007/s10567-022-00395-3

In this article the author name 'Su Yeong Kim' was incorrectly written as 'Su Yeon Kim'.

The original article has been corrected.

