# Supplementary material for: System Dynamics of Cognitive Vulnerabilities and Family Support Among Latina Children and Adolescents
Source: Clin Child Fam Psychol Rev. 2022 Mar 4;25(1):131–49. doi: 10.1007/s10567-022-00395-3 (PMC8948134; doi:10.1007/s10567-022-00395-3)
Supplement: Supplementary file 1 — Supplementary file1 (DOCX 26 kb) [file 10567_2022_395_MOESM1_ESM.docx]

# Appendix A – Model Documentation

## Overview

The Developmental Transitions model of dynamics of cognitive vulnerability and family support is a “proof of concept” model where the primary purpose is to demonstrate the feasibility of using system dynamics to develop feedback theories in developmental psychology. As such, the primary criterion for a good model is whether or not the model successfully illustrates how system dynamics could be used to develop feedback theories.

The model was developed using isee Stella Architect (version 2.1) to develop an interactive interface that can be shared online via isee Exchange and analyze the influence of loops using loop scores. Loop scores are a recent innovation for measure the impact of a feedback mechanism on the overall dynamic behavior of a system.

## Reference modes

The reference modes consist of a set of hypothetical individual trajectories in cognitive vulnerabilities from 0 to 21 years of age ranging from early pre-school onset of high levels of cognitive vulnerabilities associated with the most vulnerable children in terms of develop and risk to brief, temporary episodes of elevated cognitive vulnerabilities at each transition. Of particular interest in applying dynamical systems approaches in developmental psychology is understanding the underlying systems that might explain tipping points and path dependencies in individual developmental trajectories

## Model characteristics

The model has 26 variables including 2 stocks, 3 flows and 21 converters. The model as a total of 9 constants, 15 equations, and 6 graphical/table functions.

## Equation listings

The main equations are listed in alphabetical order of the variable with loop score built-in equations listed at the end. Each listing includes the variable name and equation, dimensions (UNITS), and documentation (DOCUMENT). Equations for stocks are given as difference equations along with their initialization equation. Graphical or table functions also provide a listing of the x-y coordinates of the table function.

1. Avoidant_Coping = GRAPH(Cognitive_Vulnerabilities)

   Points: (0.0, 0.0), (10.0, 10.0), (20.0, 20.0), (30.0, 30.0), (40.0, 40.0), (50.0, 50.0), (60.0, 60.0), (70.0, 70.0), (80.0, 80.0), (90.0, 90.0), (100.0, 100.0)

   UNITS: AC Units

   DOCUMENT: This is quantified on a 0 to 100 scale with 0 = no avoidant coping and 100 = the maximum level of avoidant coping in units of Avoidant Coping Units (AC Units). Avoidant coping is proportional to cognitive vulnerabilities. Avoidant coping is assumed to be an immediate and direct effect of cognitive vulnerabilities.
2. Cognitive_Vulnerabilities(t) = Cognitive_Vulnerabilities(t - dt) + (Developmental_Transitions + Developmental_Change_in_Cognitive_Vulnerabilities) * dt

   INIT Cognitive_Vulnerabilities = Initial_Cognitive_Vulnerabilities

   UNITS: CV Units

   DOCUMENT: Cognitive Vulnerabilities refer to a combination of attributional style, self, and future consequences (Mezulis et al 2009) that place a person at greater risk of depression in the face of stressful events. For the purpose of this model, cognitive vulnerabilities are grounded in hopelessness theory of depression (for a review, see . Liu et al, 2015), however, theories of depression will vary in how cognitive vulnerabilities are defined and measured. Cognitive Vulnerabilities are quantified on a 0 to 100 scale in CV Units as a placeholder for a specific scale or measurement model. A value of 0 corresponds to no cognitive vulnerabilities while a value of 100 corresponds to the maximum level of cognitive vulnerabilities. The initial value of Cognitive Vulnerabilities is set by the parameter, Initial Cognitive Vulnerabilities.
3. Cognitive_Vulnerabilities_AT = 2

   UNITS: Years

   DOCUMENT: Individual time constant determining how fast cognitive vulnerabilities increase or decrease. This is an individual trait.
4. Developmental_Change_in_Cognitive_Vulnerabilities = (Effect_of_Maladaptive_Behaviors_on_Cognitive_Vulnerabilities*
   Cognitive_Vulnerabilities/Cognitive_Vulnerabilities_AT –
   Effect_of_Family_Support_on_Cognitive_Vulnerabilities*
   Cognitive_Vulnerabilities/Cognitive_Vulnerabilities_AT) *
    (Max_Cognitive_Vulnerabilities - Cognitive_Vulnerabilities)/Max_Cognitive_Vulnerabilities

   UNITS: CV Units/Years

   DOCUMENT: This represents the net change in cognitive vulnerabilities due to endogenous individual and family dynamics. The rate of change reflects a natural individual recovery rate where cognitive vulnerabilities decay with a time constant (TC). The natural recovery rate is modified by the effects of maladaptive behaviors and family support, which are assumed to be independent in their direct effects on cognitive vulnerabilities. Hence, the effects of maladaptive behaviors and family support are additive as opposed to multiplicative.

   Since Cognitive Vulnerabilities are have an upper bound determined by Max Cognitive Vulnerabilities, an increase in Cognitive Vulnerabilities is constrained with the expression (Max_Cognitive_Vulnerabilities - Cognitive_Vulnerabilities)/Max_Cognitive_Vulnerabilities. However, a decrease in Cognitive Vulnerabilities is also likely to be impacted by high levels of Cognitive Vulnerabilities. For simplicity, the magnitude of the effect is assumed to be same for both increasing and decreasing cognitive vulnerabilities near the upper limits of cognitive vulnerabilities.
5. Developmental_Transitions = School_Transitions* (Max_Cognitive_Vulnerabilities-Cognitive_Vulnerabilities)/Max_Cognitive_Vulnerabilities

   UNITS: CV Units/Years

   DOCUMENT: This represents the exogenous shock of school transitions on cognitive vulnerabilities as stressful events. Specifically, we consider age dependent school transitions in this model. The impact of school transitions on Cognitive Vulnerabilities is treated as a pulse function that has a maximum fixed impact of 50 CV Units on the individual and is restricted so that Cognitive Vulnerabilities does not exceed the upper bound of 100 CV Units. Note that the magnitude of impact from school transitions on developmental transitions can differ as Cognitive Vulnerabilities approach the upper limit through the expression (Max_Cognitive_Vulnerabilities-Cognitive_Vulnerabilities)/Max_Cognitive_Vulnerabilities.
6. Effect_of_Family_Support_on_Cognitive_Vulnerabilities = GRAPH(Family_Support/50)

   Points: (0.000, 0.000), (0.200, 0.200), (0.400, 0.400), (0.600, 0.600), (0.800, 0.800), (1.000, 1.000), (1.200, 1.200), (1.400, 1.400), (1.600, 1.600), (1.800, 1.800), (2.000, 2.000)

   UNITS: Dimensionless

   DOCUMENT: This is the effect of family support on changes in developmental changes in cognitive vulnerabilities. The present formulation assumes a simple linear relationship normalized by a reference value of 50 on a 0 to 100 scale. A value of 1.0 has no effect while a value of 1.2 increases the natural rate of decreasing cognitive vulnerabilities by 20%.
7. Effect_of_Maladaptive_Behaviors_on_Cognitive_Vulnerabilities = GRAPH(Maladaptive_Behaviors/50)

   Points: (0.000, 0.000), (0.200, 0.200), (0.400, 0.400), (0.600, 0.600), (0.800, 0.800), (1.000, 1.000), (1.200, 1.200), (1.400, 1.400), (1.600, 1.600), (1.800, 1.800), (2.000, 2.000)

   UNITS: Dimensionless

   DOCUMENT: This is the effect of on changes in developmental changes in cognitive vulnerabilities. The present formulation assumes a simple linear relationship normalized by a reference value of 50 on a 0 to 100 scale. A value of 1.0 has no effect while a value of 1.2 increases vulnerabilities by 20%.
8. Family_Response_Delay = 25

   UNITS: Years

   DOCUMENT: Average perception delay in years between a change in and individual’s behavior and family response.
9. Family_Response_to_Avoidant_Coping = GRAPH(SMTH1(Avoidant_Coping/50, Family_Response_Delay))

   Points: (0.000, 0.000), (0.200, 0.100), (0.400, 0.200), (0.600, 0.300), (0.800, 0.400), (1.000, 0.500), (1.200, 0.600), (1.400, 0.700), (1.600, 0.800), (1.800, 0.900), (2.000, 1.000)

   UNITS: Dimensionless

   DOCUMENT: Response refers to the family’s recognition of changes in an individual's maladaptive behavior. This is the delayed recognition of avoidant coping. The present formulation assumes a first order information delay where there is an immediate, but delayed reaction to a change.
10. Family_Response_to_Maladaptive_Behavior = GRAPH(SMTH1(Maladaptive_Behaviors/50, Family_Response_Delay))

    Points: (0.000, 0.000), (0.200, 0.100), (0.400, 0.200), (0.600, 0.300), (0.800, 0.400), (1.000, 0.500), (1.200, 0.600), (1.400, 0.700), (1.600, 0.800), (1.800, 0.900), (2.000, 1.000)

    UNITS: Dimensionless

    DOCUMENT: Response refers to the recognition of changes in an individual's maladaptive behavior. This is the delayed recognition of maladaptive behaviors. The present formulation assumes a first order information delay where there is an immediate, but delayed reaction to a change.
11. Family_Support(t) = Family_Support(t - dt) + (Net_Change_in_Family_Support) * dt

    INIT Family_Support = Initial_Family_Support

    UNITS: FS Units

    DOCUMENT: Family support refers to overall family support of the individual with respect to changes in maladaptive and avoidant behavior and quantified on a scale of 0 to 100 Family Support Units with 0 = no family support and 100 = the maximum level of family support.
12. Family_Support_AT = 25

    UNITS: Years

    DOCUMENT: Family time constant determining how fast family support increase or decrease. This is family trait.
13. Initial_Cognitive_Vulnerabilities = 25

    UNITS: CV Units

    DOCUMENT: Initial value for cognitive vulnerabilities for an individual at birth.
14. Initial_Family_Support = 40

    UNITS: FS Units

DOCUMENT: Initial value of family support at age 0.

1. Magnitude_of_Developmental_Shocks = 50

   UNITS: CV Units

   DOCUMENT: Magnitude of shocks from school developmental transitions. These are assumed to be uniform across all developmental transitions and exogenous. However, these assumptions can be relaxed to consider more complex feedback theories.
2. Maladaptive_Behaviors = GRAPH(SMTH3(Avoidant_Coping, Onset_of_Maladaptive_Behaviors_Delay))

   Points: (0.0, 0.0), (10.0, 10.0), (20.0, 20.0), (30.0, 30.0), (40.0, 40.0), (50.0, 50.0), (60.0, 60.0), (70.0, 70.0), (80.0, 80.0), (90.0, 90.0), (100.0, 100.0)

   UNITS: MB Units

   DOCUMENT: Maladaptive Behaviors is quantified on a 0 to 100 scale with 0 = no maladaptive behaviors and 100 = the maximum level of maladaptive behavior in units of Maladaptive Behavior Units. Maladaptive Behaviors is proportional to Avoidant Coping, but has a delayed response to changes in Avoidant Coping. The assumption is that this is a third order delay where an initial period of avoidant coping will not impact the onset of maladaptive behaviors, but over time, maladaptive behaviors and has an average time of onset of MB Delay.
3. Max_Cognitive_Vulnerabilities = 100

   UNITS: CV Units

   DOCUMENT: Upper bound on cognitive vulnerabilities.
4. Max_Family_Support = 100

   UNITS: FS Units

   DOCUMENT: Upper bound on family support.
5. Net_Change_in_Family_Support = (Max_Family_Support*Family_Response_to_Maladaptive_Behavior + Max_Family_Support*Family_Response_to_Avoidant_Coping - Family_Support)/Family_Support_AT* (Max_Family_Support - Family_Support)/Max_Family_Support

   UNITS: FS Units/Years

   DOCUMENT: Changes in family support are a function of family's recognition and response to maladaptive behaviors and avoidant coping. Family support increases and decreases according to adjustment time (i.e., time constant), and moderated when family support is near the upper bound. Family support increases and decreases independently for maladaptive behaviors and avoidant coping, i.e., additive.
6. Onset_of_Maladaptive_Behaviors_Delay = 0.15

   UNITS: Years

   DOCUMENT: Average delay between changes in avoidant coping and changes in maladaptive behavior.
7. School_Transitions = PULSE(Magnitude_of_Developmental_Shocks, 5, 0) + PULSE(Magnitude_of_Developmental_Shocks, 11, 0) + PULSE(Magnitude_of_Developmental_Shocks, 14, 0) + PULSE(Magnitude_of_Developmental_Shocks, 18, 0)

   UNITS: CV Units/Years

   DOCUMENT: School transitions are represented as age specific pulse functions at ages 5, 11, 14, and 18 corresponding to school transitions into kindergarten, middle school, high school, and graduation from high school. Using a pulse function introduces a discontinuity in the simulation that could potentially generate artifacts from the numerical simulation. However, this effect can readily be checked by smoothing the pulse functions using a first or third order delay. This would conceptually correspond to a theory where the direct effects of school transitions on cognitive vulnerabilities were spread out over time. While this may be more realistic, the smoothing of the transitions also makes it harder to distinguish the exogenous school transitions effects from the endogenous individual and family response effects. Hence, for simplicity of analysis, school transitions have been modeled as pulse functions.

## Simulation specifications

| Run Specs | |
| --- | --- |
| Start Time | 0 |
| Stop Time | 21 |
| DT | 1/1024 |
| Fractional DT | True |
| Save Interval | 0.0009765625 |
| Sim Duration | 0 |
| Time Units | Years |
| Pause Interval | 0 |
| Integration Method | RK4 |
| Track flow quantities | True |
| Keep all variable results | True |
| Run By | Run |
| Calculate loop dominance information | True |
| Exhaustive Search Threshold | 1000 |

## Parameters and initial conditions for each scenario

| Case | *Cognitive Vulnerabilities AT* | *Family Response Delay* | *Family Support AT* | *Initial Cognitive Vulnerabilities* | *Initial Family Support* | *Onset of Maladaptive Behaviors Delay* |
| --- | --- | --- | --- | --- | --- | --- |
| 1 | 0.125 | 0.125 | 0.125 | 25 | 20 | 0.25 |
| 2 | 0.5 | 25 | 25 | 25 | 20 | 0.125 |
| 3 | 1.25 | 0.1 | 12 | 25 | 20 | 0.5 |
| 4 | 2 | 25 | 25 | 25 | 55 | 0.15 |
| 5 | 2 | 25 | 25 | 25 | 40 | 0.15 |
| 6 | 2 | 25 | 25 | 25 | 45 | 0.15 |

## Known issues and limitations

There are a number of known issues and limitations of the current version of the model. This section lists the currently known issues and limitations. It is important to stress that these are a general list of limitations, that is, not specific to a “proof of concept” simulation model. For a proof-of-concept simulation model, the model can still meet the criteria for a good proof-of-concept model because the main purpose is to illustrate the feasibility of an approach, and *not to provide a “complete” model* from applying the approach.

Some limitations are introduced as a matter of convenience in developing a proof-of-concept model, while others are issues that call for further consideration and refinement in subsequent work. The determination as to whether or not a limitation needs to be addressed for a proof-of-concept model to be successful depends on whether or not the model is “good enough” to demonstrate the feasibility of the approach. For example, a limitation that the model isn’t grounded in data can be fair, but whether or not the critique applies to the proof-of-concept model depends how grounding the proof-of-concept model will enhance the feasibility of the approach. Certainly, grounding a proof-of-concept model in data would strengthen the feasibility, but would addressing that limitation be a prerequisite for a project or would the limitation serve as sufficiently clear to motivate funding for a project?

Other limitations are more along the lines of psychological theory. For example, a concept like cognitive vulnerabilities origins in a set of psychological theories, which vary in how they define and use the concept quantitatively. In this sense, choosing one definition over another could represent a limitation. However, the purpose of this proof-of-concept model is not take a particular position in arguing for one theory over another, but instead to illustrate how a theory can be represented in system dynamics and used to develop a theory. There could also be limitations in psychological theory that stem from modeling assumptions lacking any form of scientific justification in terms of psychological theory. This could be, at best, distracting with respect to demonstrating the feasibility of an approach, and at worst, illustrate some very real issues from a meta-theoretical level that would need to be addressed within a proof-of-concept model within a specific domain.

That said, the model has the following known issues and limitations.

1. ***Family support is assumed to always be effective in the beneficial direction.*** While the model allows for families to vary in their initial level of support, how fast they recognize an issue, and how fast they can rally support, support is always assumed be effective. This is clearly wrong. Oftentimes, families do not know what will be helpful or what may be helpful for one child or adolescent may be ineffective or harmful for another. Moreover, there are many situations—often of greatest clinical concern—where a family member or family is outrightly neglecting or harming a child or adolescent, not as a misguided strategy of parenting, but as a consequence of the severe limitations in resources or exploitation.
2. ***Developmental change is assumed to be symmetric with respect to increasing or decreasing cognitive vulnerabilities near the maximum value.*** The model assumes that endogenously driven increases in cognitive vulnerabilities near the maximum value happen at the same rate as decreases. This is probably incorrect since the mechanisms of recovering from cognitive vulnerabilities is unlikely to be the same as the mechanism contributing to cognitive vulnerabilities, i.e., an individual is more likely to develop cognitive vulnerabilities fast than they can recover from them.
3. ***Cognitive vulnerabilities do not change when the stock is at the maximum value.*** When cognitive vulnerabilities = 100, there is not a way for cognitive vulnerabilities to decrease and cognitive vulnerabilities is in dynamic equilibrium. However, the asymptotic behavior is different. In some scenarios very close to the level of cognitive vulnerabilities = 100, there are a rich set of underlying dynamics.
